# Molecular docking analysis of nitisinone with homogentisate 1,2 dioxygenase

**DOI:** 10.6026/97320630013136

**Published:** 2017-05-31

**Authors:** Narges Zolfaghari

**Affiliations:** 1 National institute of genetic engineering and biotechnology, Tehran, Iran

**Keywords:** Alkaptonuria, Lipinski rules, drug design

## Abstract

Alkaptonuria is an inherited disease that is caused by homogenticate accumulation. Deficiency or mutation in Homogentisate 1,2 dioxygenase gene (chromosome 3q21-q23) leads to production of incorrectly folded or truncated enzyme. Several studies indicated that competitive inhibitors of Homogentisate 1,2 dioxygenase like Nitisinone could be used for Alkaptonuria treatment. Therefore, it is of interest to design better inhibitors of the enzyme. We used subset 3_p.0.5 from Zinc database as the virtual screening library by PyRx software relaying on Lamarckian genetics algorithm. Top 10 hits with more efficient binding affinity were analyzed and hit No#5 and No# 7 was selected for further design. In Lig No#5, we decreased the hydrophobicity by adding oxygen in the hydrophobic tail of the molecule at positions C5 and C10. The new compound of (2Z, 5Z, 8Z)-6,9-Dihydroxy-2-(2-hydroxy-5-oxo-1,3-cyclohexadien-1-yl)-2,5,8-decatrienoic acid satisfied Lipinski rules as well as PhysChem and FafDrugs filters. Moreover, the modified version of Lig No# 7 with the IUPAC name of [2-(Carboxymethyl)-3,5-dihydroxyphenyl] acetic acid satisfies the Lipisnki, FafDrugs and Physchem.

## Background

Tyrosine (4-hydroxyphenylalanine) pathway is a key pathway. It is the precursor of several vital chemicals. Tyrosine is the precursor of dopamine, norepinephrine and epinephrine [1,2]. In addition, several alkaloid and pigments are derived from tyrosine [3,4,5]. Therefore, any misfunction within the pathway would lead to clinical symptom. Several enzymes are engaged in tyrosine degradation. First, tyrosine amino transferase converts it to 4- hydroxyphenyl pyruvic acid. Then hydroxyphenylpyruvate dioxygenase produces homogenticic acid, a precursor of maleylacetoacetic acid. Several genetic disorders are associated with the tyrosine pathway and Alkaptonuria is common among them. It is caused by defect in homogentisate dioxygenase leading to homogentisic acid accumulation [6]. Nitisinone (2-[2-nitro-4- (trifluoromethyl)benzoyl]cyclohexane-1,3-dione), a triketone is used for treating inherited Alkaptonuria. In a study on tyrosine pathway, Tinti, L. et al found that antioxidant N-acetylcysteine can be used for treatment of Alkaptonuria patients [7]. In another study, 3-Cyclopropanecarbonyloxy-2-cyclohexen-1-one reported as a potent non-triketone type inhibitor of 4-hydroxyphenylpyruvate dioxygenase [8]. The carbonyl groups within the identified structure were reported to be crucial in the binding efficiency. Both N-acetylcysteine and Cyclopropanecarbonyloxy-2-cyclohexen-1- one are Hydroxyphenylpyruvate dioxygenase inhibitors, which by binding to the active site of enzyme and making steric condense, inhibit catalysis in a competitive manner. As the results, 2,5- dihydroxyphenylacetic acid (homogentisic acid) production rate would be decreased. The most clinical signs of alkaptonuria are related to homogentisic acid accumulation and its polymerization. Therefore, by decreasing the homogentisic acid levels, the clinical signs would be decreased. At the present study, we are trying to introduce new chemicals with Lipinski and FafDrugs standards. For gaining this purpose, we used several tools that mostly designed based on artificial neural networks connected to experimentally approved wet lab data. Also for decreasing the flexibility of the molecule, we have changed three single bonds to double, one at position C4 and the other at C9 and C20.

## Methodology

### Drug target and ligands

We used the Crystal structure of human 4-Hydroxyphenylpyruvate dioxygenase From Protein Data Bank (PDB) database (http://www.rcsb.org/pdb/home/home.do) with PDB code of 3isq. Homogentisic acid and Nitisinone structures were retrieved from Pubchem database (https://pubchem.ncbi.nlm.nih.gov/) by pubchem ID of CID_870 and CID_115355 respectively. Virtual screening library was retrieved from Zinc database.

### Molecular ducking and pharmacological analysis

A drug like category subset from Zinc database (3_p.0.5) was downloaded and used as the ligand database for virtual screening purpose [9]. Molecular docking operation was carried out by PyRx software [10], which is a GUI tool, based on AutoDock [11]. In the next step, we compared the affinity binding energies and selected top inhibitors that indicated the highest interactions with our macromolecule. In order to modify the top hits, we used HyperChem software. The modifications performed in a way that modified ligands improve in drug likeness properties and binding affinity. Vega ZZ and OpernBable GUI tools were also used to optimize geometry and format conversion. Finally, the rationally designed ligands checked by FAFDrugs3 web server (http://fafdrugs3.mti.univ-paris-diderot.fr/) for analyzing changes in the pharmacological properties, which is caused by ligand modification. Based on FAFDrugs3 errors, we edited the structures.

## Results and discussion

Formation of Homogentisic polymers is pathogenic. It is caused by mutation in Homogentisate 1,2 dioxygenase gene located in 3q21- q23, which leads to accumulation of homogentisic acid (HGA). High levels of HGA an intermediary metabolic in tyrosine and phenylalanine pathway, causes several diagnostic signs including black color of urine due to oxidation or alkalization. In addition, oxidized HGA gather and form polymeric structures, which its deposition in connective tissue leads to chronic pigmentation of skin and degenerative arthritis. In order to prevent HGA accumulation, we have targeted the upstream enzyme, which is responsible for HGA production. Partial inhibition of Homogentisate 1,2 dioxygenase leads to less HGA production. Therefore, we have designed several chemicals, which theoretically could reversibly bind to the active site of the enzyme. In order to find the active site of Hydroxyphenylpyruvate dioxygenase we have used molecular docking approach by two experimentally approved ligands: (I) the homogenticiate as the natural ligands and (II) the Nitisinone [12], commercial drug that acts as a competitive inhibitor. The docking process was carried out with a radius of 38 Å to cover the entire structure of the enzyme. All of the generated poses for both homogenticate and Nitisinone attached to the main cavity of the enzyme with coordinates of X: -0.12, Y: 45.26 and Z: 4.09. The radius of ligands steric condense was six Å. We have used the found coordinate with a bigger radius of eight for virtual screening and rational drug design purpose.

Among 100,000 drugs like chemicals, top 10 successive hits with the most negative binding affinity were selected for further drug design purpose. Table 1 describes the binding affinity as well as pharmacological properties of top virtual screening hits. Ligand No# 5 and Ligand No# 7 were indicated best binding affinity of -9.6 and -9.1 and selected for further modifications to reach a theoretical drug structure. To do this, first lig#5 was checked from pharmacological aspect by Lipinski rules and FAFDrugs3 filters including: Pan Assay interference compound (PAINS) A, B and C with hydrophobicity calculation method of XLOGP. The molecule was rejected by the following errors: High_Risk_consecutive_alkyl_chains, High_Risk_michael_acceptors, Low_Risk_para_hydroquinone, Covalent_michael_acceptors, Covalent_alpha_beta_unsaturated_carbonyl, and LogP. Moreover, the rotatable bonds were eight and the structural flexibility was high. It could generate so many conformations that can bind to many molecules in the body. To perform structural modification, we have reduced hydrophobicity by adding oxygen in the hydrophobic tail at positions C5 and C10. Also for decreasing the flexibility of the molecule, we have changed two single bonds to double, one between C4 and C5 and the other one between C9 and C10. To do this, the saturated Carbons were changed to unsaturated form by removing extra hydrogens. Molecular dynamics simulation was performed in the modified structure by MM+ force field for 50 Ps to reach the optimal conformation. The modified ligand (RD-5-1) was re-analyzed regarding binding affinity and pharmacological properties. Interestingly, the pharmacological properties was significantly improved and could pass the Lipinski rules as well as other pharmacological properties which provided by FafDrugs3 like PhysChem and just the number of Hydrogen bond donors were remained as the error. By unsaturation of C20 in the ring and making a double C=O bond, the flexibility decreased more and the number of hydrogen donors reduced. Finally, the molecule RD-5-2 could reach the lead like standards. Moreover molecular dynamics simulation for 50 PS was performed in RD-5-2 by MM+ force field; the binding affinity of RD-5-2 was significantly increased to -9.7. In other hands, Ligand No# 7 with a binding affinity of -113.931 could fit to PhysChem standards but due to Low_Risk_para_hydroquinone error, it ranked as an intermediate molecule in FafDrugs3 filters. By changing the position of O1 from O1-C11 to O1-C13, the para hydroquinone structure changed to Orto hydroquinone. The new modified molecule (RD-7-1) was re-analyzed regarding binding affinity and indicated a less decrease in the binding efficiency by the score of -8.8.

## Conclusion

We have calculated the binding affinity of Nitisinone to Hydroxyphenylpyruvate dioxygenase as -9.3. Nitisinone, RD-5-2 and RD-7-1, indicated similar binding affinities. Moreover, both RD-5-2 and RD-7-1 satisfied Lipinski rules and PhysChem filters. Thus, RD-5-2 and RD-7-1 are potential inhibitors to the enzyme.

## Figures and Tables

**Table 1:** The pharmacological properties and binding affinity of successive hits from virtual screening among 100,000 ligands retrieved from subset 3_p.0.5 Zinc database against the active site of human homosentisate dioxygenase in the coordinates of X: -0.12, Y: 45.26 and Z: 4.09. The properties used are PhysChem and FAFDrugs filters. HBD: hydrogen bonds donor. HBA: hydrogen bond acceptor. tPSA: topological polar surface area. Logsw: Estimating Aqueous Solubility Directly from Molecular Structure calculated by ESOL algorithm. LogD: pharmacological property of drug likeness with respect to absorption from the intestine. LogP : indicator of hydrophobicity.

Ligand	Smiles	Binding	MW	logP	logD	logSw	tPSA	RotatableB	RigidB	Flexibility	HBD	HBA	HBD_HBA	TotalCharge	ratioH/C
1	OC(=O)C(=C)c1cc(O)ccc1O	-8.34	180.16	1.31	-1.76	-1.86	80.59	2	8	0.2	3	4	7	-1	0.44
2	CCOC(=O)Cc1cc(O)ccc1O	-8.45	196.2	1.55	1.5	-1.94	66.76	4	7	0.36	2	4	6	0	0.4
3	OC(=O)CCc1cc(O)ccc1O	-8.87	182.17	0.97	-1.81	-1.58	80.59	3	7	0.3	3	4	7	-1	0.44
4	C[C@@H](C(O)=O)c1cc(O)ccc1O	-8.98	182.17	1.25	-1.77	-1.82	80.59	2	7	0.22	3	4	7	-1	0.44
5	CCCC[C@@H](C(O)=O)c1cc(O)ccc1O	-9.6	224.25	2.69	-0.28	-2.73	80.59	5	7	0.42	3	4	7	-1	0.33
6	CCCCCCC\C=C(\C(O)=O)c1cc(O)ccc1O	-8.67	278.34	4.68	1.54	-4.09	80.59	8	8	0.5	3	4	7	-1	0.25
7	COC(=O)CCc1cc(O)ccc1O	-9.1	196.2	1.3	1.59	-1.78	66.76	4	7	0.36	2	4	6	0	0.4
8	OC(=O)C(=O)Cc1cc(O)ccc1O	-8.88	196.16	0.74	-2.22	-1.53	97.66	3	8	0.27	3	5	8	-1	0.56
9	OC(=O)Cc1c(O)ccc(O)c1CC(O)=O	-8.65	226.18	0.14	-6.07	-1.24	120.72	4	8	0.33	4	6	10	-2	0.6
10	OC(=O)Cc1c(O)ccc(O)c1F	-8.07	186.14	0.77	-2.33	-1.57	80.59	2	7	0.22	3	4	7	-1	0.63

**Figure 1:**
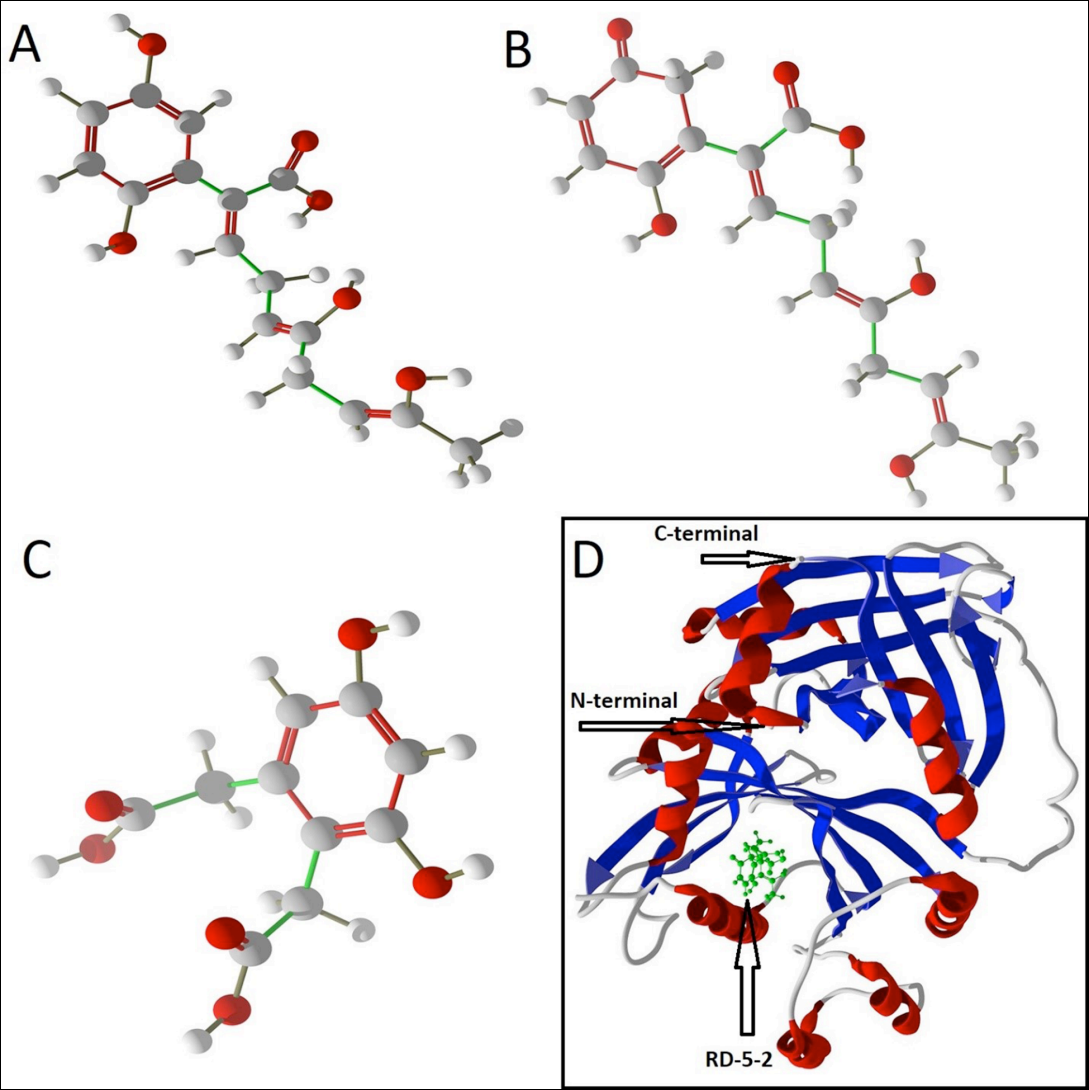
A: The structure of rationally designed ligand RD-5-1 that is the modified version of Ligand 5 (The successive hit No# 5). B: The structure of rationally designed ligand RD-5-2. C: The structure of rationally designed ligand RD-7-1 that is the modified version of Ligand 7. D: RD-5-2 in the active site of homogentisate dioxygenase with the coordinates of X: -0.12, Y: 45.26 and Z: 4.09.
